# The Prognostic Value of Lung Ultrasound in Patients With Newly Diagnosed Heart Failure With Preserved Ejection Fraction in the Ambulatory Setting

**DOI:** 10.3389/fcvm.2021.758147

**Published:** 2021-12-02

**Authors:** Blanka Morvai-Illés, Nóra Polestyuk-Németh, István Adorján Szabó, Magdolna Monoki, Luna Gargani, Eugenio Picano, Albert Varga, Gergely Ágoston

**Affiliations:** ^1^Department of Family Medicine, Albert Szent-Györgyi Medical School, University of Szeged, Szeged, Hungary; ^2^Emergency Patient Care Unit, Albert Szent-Györgyi Medical School, University of Szeged, Szeged, Hungary; ^3^Mures County Clinical Hospital, Cardiology Department, George Emil Palade University of Medicine, Pharmacy, Science, and Technology of Targu Mures, Targu Mures, Romania; ^4^Institute of Clinical Physiology, National Research Council, Pisa, Italy

**Keywords:** heart failure with preserved ejection fraction (HFpEF), diagnosis, lung ultrasonography (LUS), echocardiography, prognosis

## Abstract

**Background:** Heart failure with preserved ejection fraction (HFpEF) is a growing healthcare burden, and its prevalence is steadily increasing. Lung ultrasound (LUS) is a promising screening and prognostic tool in the heart failure population. However, more information on its value in predicting outcome is needed.

**Aims:** The aim of our study was to assess the prognostic performance of LUS B-lines compared to traditional and novel clinical and echocardiographic parameters and natriuretic peptide levels in patients with newly diagnosed HFpEF in an ambulatory setting.

**Methods:** In our prospective cohort study, all ambulatory patients with clinical suspicion of HFpEF underwent comprehensive echocardiography, lung ultrasound and NT-proBNP measurement during their first appointment at our cardiology outpatient clinic. Our endpoint was a composite of worsening heart failure symptoms requiring hospitalization or loop diuretic dose escalation and death.

**Results:** We prospectively enrolled 75 consecutive patients with HFpEF who matched our inclusion and exclusion criteria. We detected 11 events on a 26 ± 10-months follow-up. We found that the predictive value of B-lines is similar to the predictive value of NT-proBNP (AUC 0.863 vs. 0.859), with the best cut-off at >15 B-lines. Having more B-lines than 15 significantly increased the likelihood of adverse events with a hazard ratio of 20.956 (*p* = 0.004). The number of B-lines remained an independent predictor of events at multivariate modeling. Having more than 15 B-lines lines was associated with a significantly worse event-free survival (Log-rank: 16.804, *p* < 0.001).

**Conclusion:** The number of B-lines seems to be an independent prognostic factor for adverse outcomes in HFpEF. Since it is an easy-to-learn, feasible and radiation-free method, it may add substantial value to the commonly used diagnostic and risk stratification models.

## Introduction

Heart failure with preserved ejection fraction (HFpEF) already makes ~50% of heart failure patients. Since the prevalence of its common risk factors is rising, HFpEF is expected to be diagnosed more often (1). Although its prognosis is considered better than that of HF with reduced ejection fraction (HFrEF), both the mortality and hospitalization rates are very high (1, 2). HFpEF is a heterogeneous, multifactorial disease. The diagnosis is often challenging; therefore, several score systems have been devised to facilitate the diagnosis and assess the prognosis. The score systems were mainly validated on the hospitalized and acute HFpEF population (3–5). Imaging parameters are included in the H2FPEF and HFA-PEFF scores, designed initially as diagnostic score systems. A recent study based on more than 900 HFpEF patients could not validate their prognostic utility (6). The diagnostic use of NT-proBNP in HFpEF is well-established (7), and the data are convincing about its predictive value (8). However, a number of studies suggested that its prognostic value remains controversial (9–11).

A common abnormality in HFpEF is elevated left ventricular (LV) filling pressure, leading to elevated left atrial (LA) pressures and, eventually, to the development of pulmonary congestion (PC) (12). PC is a universal finding in HF and implies a higher risk for hospitalization and death in both acute and chronic HF (13). Through B-line evaluation, lung ultrasound (LUS) has been recently proposed as a simple, radiation-free, non-invasive tool to assess PC (14, 15). The number of B-lines is related to pulmonary capillary wedge pressure (16), NT-proBNP (17), and E/e′ in HF patients (18). LUS has a prognostic value in acute HF irrespective of EF (19) and chronic HF regardless of EF (20, 21).

We aimed to assess the prognostic value of B-lines and other novel ultrasound parameters (such as global longitudinal strain and left atrial reservoir strain) in newly diagnosed HFpEF patients.

## Materials and Methods

### Study Population

One hundred and thirty-one consecutive patients were screened at our cardiology outpatient clinic (University of Szeged, Hungary) between January 2018 and December 2019. General practitioners referred all patients with mild or moderate HF symptoms. None of the patients had a previous diagnosis of HF. Data collection was based on a standardized clinical questionnaire performed by a researcher blinded to clinical records. Our inclusion criteria were: (1) age ≥ 18 years; (2) diagnosis of HFpEF defined in the 2016 ESC guideline (22); (3) absence of atrial fibrillation with > 80/min at rest; (4) no prior history of the following: interstitial lung disease, moderate or severe COPD (Chronic Obstructive Pulmonary Disease), bronchial asthma or pulmonary hypertension; (5) absence of moderate or severe aortic or mitral valve disease on the screening echocardiogram; (6) no history of cardiomyopathies; (7) absence of severe kidney failure or anemia (eGFR ≥ 35 ml/min, Hgb ≥ 100 g/l); absence of malignancy (except localized basal cell carcinoma of the skin or localized prostate cancer). Data handling and publication respected the Declaration of Helsinki. The registration number of ethical approval is 131/2018/SZTE.

### Ultrasound Assessment

**Figure 1:**
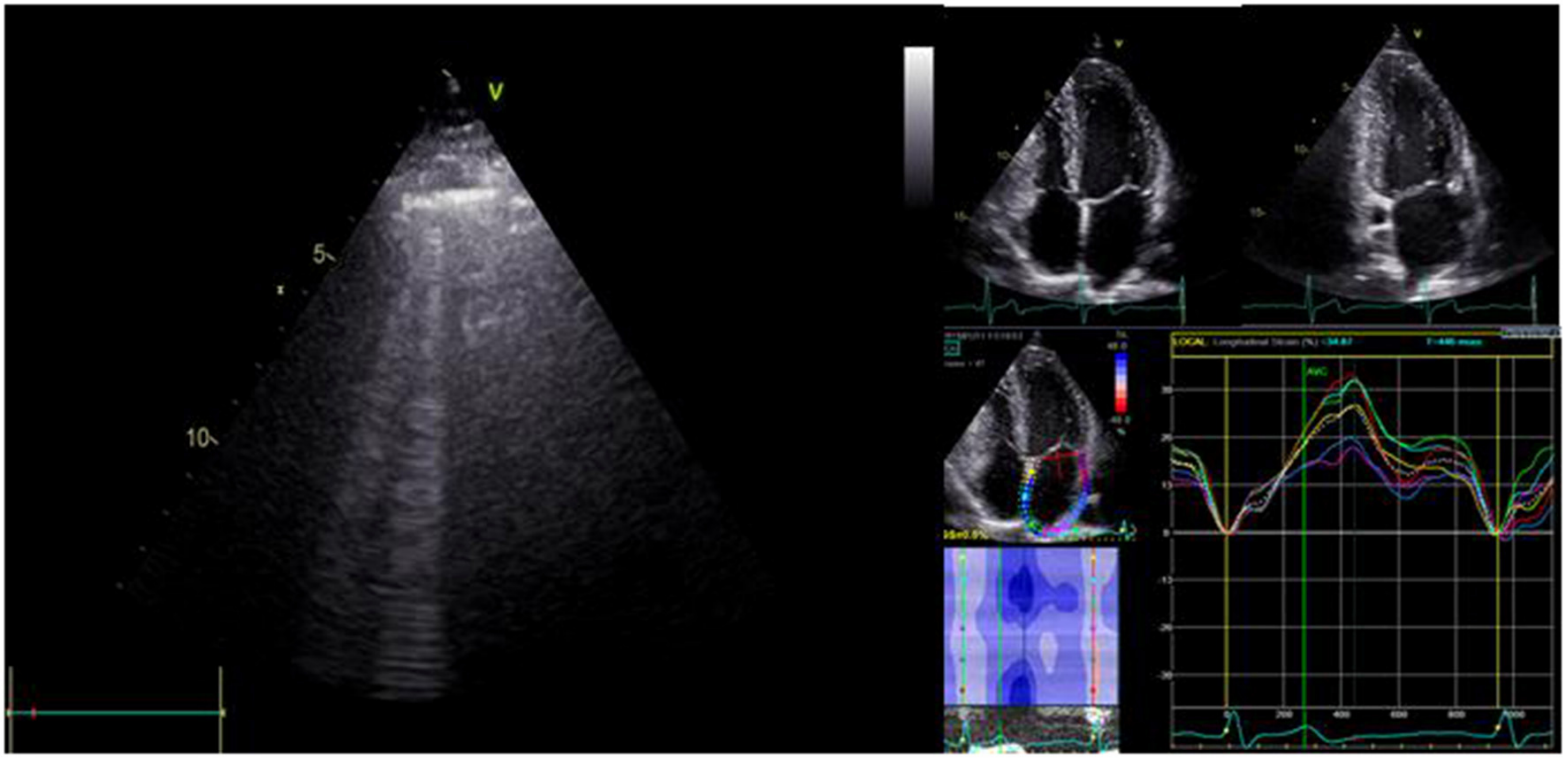
Assessment of B-lines by lung ultrasound and determination of left atrial reservoir strain (LASr) in patients with HFpEF.

A comprehensive transthoracic echocardiogram (TTE) was performed using a Vivid-S70 (GE Vingmed, Horten, Norway) ultrasound machine equipped with the 3S probe (1.5–3.6 MHz). An experienced cardiologist with EACVI-TTE certification performed all measurements according to the recommendations of the American Society of Echocardiography and the European Association of Cardiovascular Imaging (23, 24). Myocardial deformation was analyzed with GE EchoPAC (version v202) software. LV strain was measured according to EACVI recommendations (25). QRS complex was used as a time reference. LA strain parameters were recorded as per the EACVI consensus document and were *post hoc* analyzed by two experienced physicians (26). ECG trigger was used as a time reference, using the upslope of the R wave as a surrogate of end-diastole. In case of any uncertainty, the strain pattern itself provided support (and mitral inflow pattern in patients with sinus rhythm). From apical four- and two-chamber views with a frame rate of 40–80 frames per second, three consecutive cardiac cycles were acquired and averaged in each patient. Region of Interest (ROI) was defined by using a point-and-click approach for tracking the endocardial border. Longitudinal strains were calculated, defined as strain in the direction tangential to the endocardial atrial border. Strain curves during reservoir phase were evaluated (Figure 1).

Immediately after transthoracic echocardiography, patients underwent LUS performed by the same cardiologist, who obtained the echocardiographic measurements to assess B-lines using the same probe and echocardiography machine. We screened the anterior and lateral hemithoraces, scanning along the parasternal, midclavicular, anterior axillary and midaxillary lines from the second to the fifth intercostal space on the right hemithorax and the second to the fourth intercostal space on the left, adding up to a total of 28 zones (27). A B-line was defined as a discrete, comet-like vertical hyperechoic reverberation artifact starting from the pleural line, extending to the bottom of the screen and moving synchronously with lung sliding (28). The operator, with dedicated training and previous experience in LUS, acquired and analyzed all LUS studies and was blind to the NT-proBNP value.

### NT-proBNP

Within 1 hour of the cardiac and lung ultrasound, peripheral venous blood samples were obtained from each patient. NT-proBNP analysis was performed using the Elecsys 2010 analyzer (Roche Diagnostics, Mannheim, Germany).

### Follow Up Data

Follow-up data were collected every 3 months *via* phone calls to monitor clinical status and adverse outcomes. Outpatient visits were performed 6-monthly when clinical status and adverse events were recorded. A composite HF endpoint was created, including death (any cause), hospitalization for acute decompensation of HF, and worsening HF (defined as the intensification of loop diuretic therapy). Information about the endpoint events were retrieved from medical records.

### Statistical Analysis

Our data are expressed as number and percentage for categorical and mean ± standard deviation, or median for continuous variables. Univariate comparisons were made by chi-square or independent samples *T*-test, as appropriate. A *p*-value < 0.05 was accepted as statistically significant. Receiver-operating characteristic (ROC) curves were used to compare the predictive value of B-lines and NT-proBNP for the composite endpoint. The corresponding area under the curves (AUC) was reported. The correlations between NT-proBNP and other parameters were analyzed with Spearman correlation. Univariate and multivariate (Backward LR method) Cox regression analysis was used to assess the prognostic capacity of parameters. Collinearity had been excluded using variance inflation factor <3 before the analysis. Results were reported as Hazard Ratios. Event-free survival was calculated using Kaplan-Meier curves and the log-rank test to determine significance between groups. Data were analyzed using IBM SPSS 22 statistical software.

## Results

One hundred and thirty-one consecutive patients were screened from January 2018 to December 2019. Fifty-six patients were excluded (14 patients had moderate or severe mitral and/or aortic valve disease, 2 patients had atrial fibrillation with heart rate above 80/min at rest, 10 patients had an EF below 50%, 4 patients had moderate or severe COPD or pulmonary disease, 2 patients had eGFR below 35 mL/min/1.73 m^2^, 3 patients had ischemic heart disease, where subsequent examinations were confirming significant coronary artery disease). In 21 patients, we could not confirm any significant disorder that could support the referral diagnosis. Finally, 75 patients (age: 70.33 ± 6.85, 73.3% female) met our inclusion criteria. Ten patients had atrial fibrillation with normal ventricular rate during the enrollment, and others were in sinus rhythm. Patient characteristics are shown in Table 1. Patients with adverse clinical events more frequently had hyperlipidemia, diabetes mellitus, ongoing digoxin therapy, higher NT-proBNP levels, more B-lines, lower LASr, DCT and S' velocity than the event-free group.

**Table 1:** Baseline demographic and echocardiographic parameters.

**Parameters**	**Overall ***n*** = 75**	**HF event free group ***n*** = 64**	**HF event group ***n*** = 11**	**Significance**
**Demographic parameters**				
Age (years)	70.33 ± 6.85	70.02 ± 7.02	72.18 ± 5.67	-
Gender (female, *n*, %)	55 (73.30%)	49 (76.56%)	6 (54.54%)	-
Body mass index (kg/m^2^)	30.15 ± 4.89	29.96 ± 4.64	31.13 ± 6.20	-
**Clinical parameters**				
Systolic blood pressure (mmHg)	134.24 ± 15.05	134.91 ± 15.60	130.33 ± 11.15	-
Diastolic blood pressure (mmHg)	79.00 ± 9.35	79.57 ± 9.70	75.67 ± 6.40	-
Heart rate (beats/min)	68.48 ± 10.55	67.65 ± 9.73	72.82 ± 13.81	-
NYHA I. (*n*, %)	3 (4%)	3 (4.69%)	0 (0%)	
NYHA II. (*n*, %)	60 (80%)	53 (82.81%)	7 (63.64%)	
NYHA III. (*n*, %)	11 (14.67%)	7 (10.77%)	4 (36.36%)	
NYHA IV. (*n*, %)	0 (0%)	0 (0%)	0 (0%)	
NT-proBNP level (pg/ml)	406.60 (165, 772)	376.95 (163, 640)	904.00 (668, 2,156)	0.01
eGFR (ml/min)	71.45 ± 17.54	73.12 ± 17.16	63.59 ± 18.32	-
Hemoglobin (g/l)	130.33 ± 14.83	130.57 ± 12.80	129.22 ± 22.80	-
**Comorbidities**				
Hypertension (*n*, %)	65 (86.67%)	56 (84.50%)	9 (81.82%)	-
Diabetes mellitus (*n*, %)	21 (28.00%)	15 (23.44%)	6 (54.54%)	0.025
Atrial fibrillation (*n*, %)	21 (28.00%)	16 (25.00%)	5 (45.45%)	-
Hyperlipidaemia (*n*, %)	27 (36.00%)	19 (29.69%)	8 (72.72%)	0.006
**Treatment**				
Beta-blocker (*n*, %)	55 (73.33%)	48 (75.00%)	7 (63.64%)	-
Angiotensin convertase enzyme inhibitor (*n*, %)	30 (40.00%)	27 (42.19%)	3 (27.27%)	-
Angiotensin receptor blocker (*n*, %)	28 (37.33%)	21 (32.81%)	7 (63.64%)	-
Calcium channel blocker (*n*, %)	20 (26.67%)	16 (25.00%)	4 (36.36%)	-
Digoxin (*n*, %)	4 (5.33%)	2 (3.12%)	2 (18.18%)	0.045
Loop diuretic (*n*, %)	44 (58.67%)	36 (56.25%)	8 (72.73%)	-
Aldosterone antagonist (*n*, %)	5 (6.67%)	4 (6.25%)	1 (9.09%)	-
Statin (*n*, %)	34 (45.33%)	27 (42.19%)	7 (63.64%)	-
Anticoagulant (*n*, %)	23 (30.67%)	18 (28.12%)	5 (45.45%)	-
Proton pump inhibitor (*n*, %)	32 (42.67%)	25 (39.01%)	7 (63.64%)	-
**Echocardiographic parameters**				
EF (%)	67.56 ± 8.32	68.92 ± 7.39	62.82 ± 6.69	0.013
LV GLS (%)	−16.67 ± 6.38	−17.21 ± 6.52	−13.26 ± 4.27	-
IVS (mm)	11.35 ± 1.30	11.30 ± 1.11	11.64 ± 2.16	-
PW (mm)	11.20 ± 1.41	11.16 ± 1.21	11.45 ± 2.34	-
LV mass index (g/m^2^)	114.22 ± 26.07	112.70 ± 22.43	112.13 ± 40.66	-
RWT	0.45 ± 0.07	0.45 ± 0.07	0.44 ± 0.08	-
LAVI (ml/m^2^)	43.85 ± 16.22	45.57 ± 16.25	43.65 ± 16.81	-
LASr (%)	19.76 ± 8.83	20.71 ± 8.84	14.46 ± 6.98	0.038
E/A	1.04 ± 0.56	1.02 ± 0.56	1.18 ± 0.52	-
DCT (ms)	223.24 ± 69.08	231.45 ± 65.42	177.30 ± 74.89	0.021
E/E′ mean	10.82 ± 3.81	10.61 ± 3.62	12.28 ± 4.93	-
S′ (cm/s)	8.41 ± 2.76	8.73 ± 2.82	6.54 ± 1.37	0.014
PASP (mmHg)	37.50 ± 14.97	36.21 ± 14.1	45.10 ± 18.34	-
TAPSE (mm)	25.14 ± 5.42	25.31 ± 5.46	24.18 ± 5.31	-
No of B-lines	11 (5, 20)	9 (4,15)	21(17, 33)	<0.001
B-lines > 30 (*n*, %)	50 (66.70%)	7 (10.94%)	3 (27.27%)	-
B-lines > 15 (*n*, %)	25 (33.30%)	15 (23.44%)	10 (90.91%)	<0.001

*NYHA, New York Heart Association classification to stages of heart failure; NT-proBNP, N-terminal (NT)-prohormone B type natriuretic peptide; eGFR, estimated Glomerular Filtration Rate; LV EF, Left Ventricular Ejection Fraction; LV GLS, Left Ventricular Global Longitudinal Strain; IVS, Inter Ventricular Septum Thickness; PW, Posterior Wall Thickness; LV mass index, Left Ventricular Mass Index; RWT, Relative Wall Thickness; LAVI, Left Atrial Volume Index; LASr, Left Atrial Reservoir Strain; DCT, E wave deceleration time; E/E′ mean, the relationship between maximal values of passive mitral inflow (E, PW-Doppler) and the average of lateral and septal early diastolic mitral annular velocities (E, TDI); TDI, Tissue Doppler Imaging; S′, systolic myocardial velocity measured with TDI; PASP, pulmonary artery systolic pressure; TAPSE, Tricuspid annular plane systolic excursion*.

**Figure 2:**
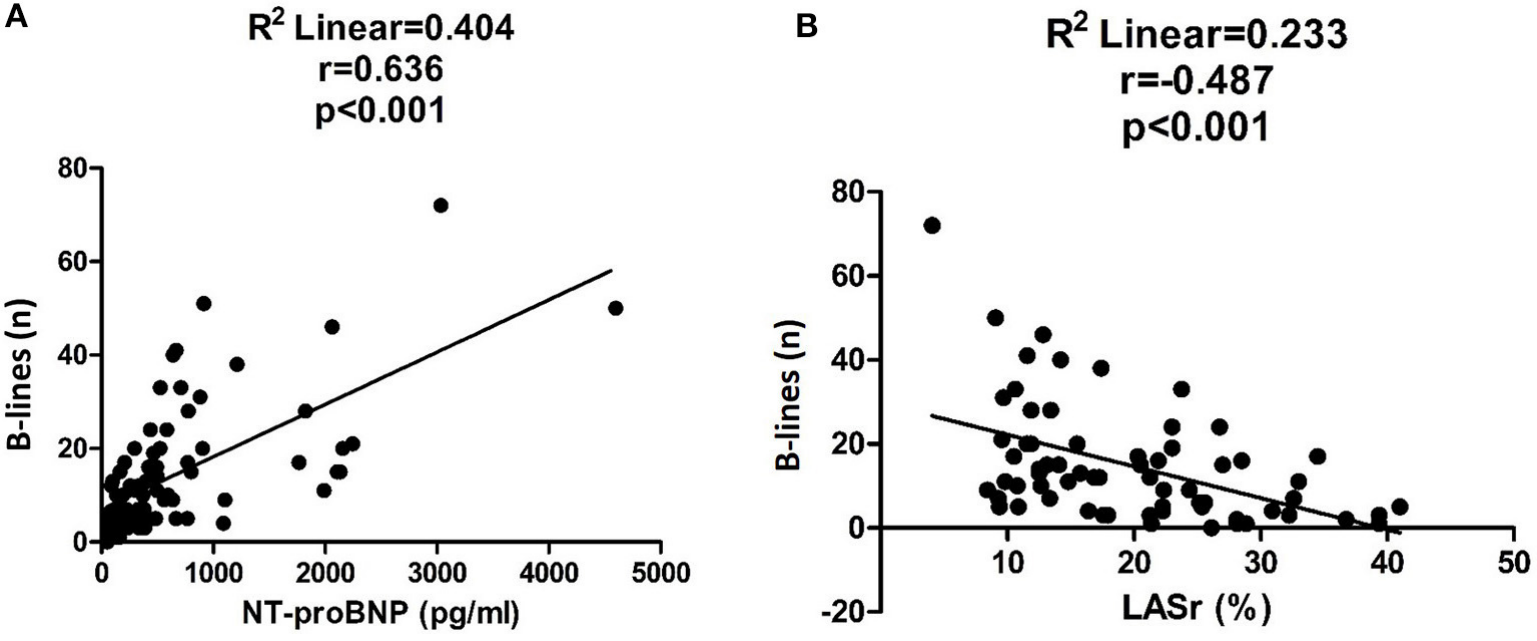
Correlation between the number of B-lines and NT-proBNP levels **(A)** and LASr values **(B)**.

**Figure 3:**
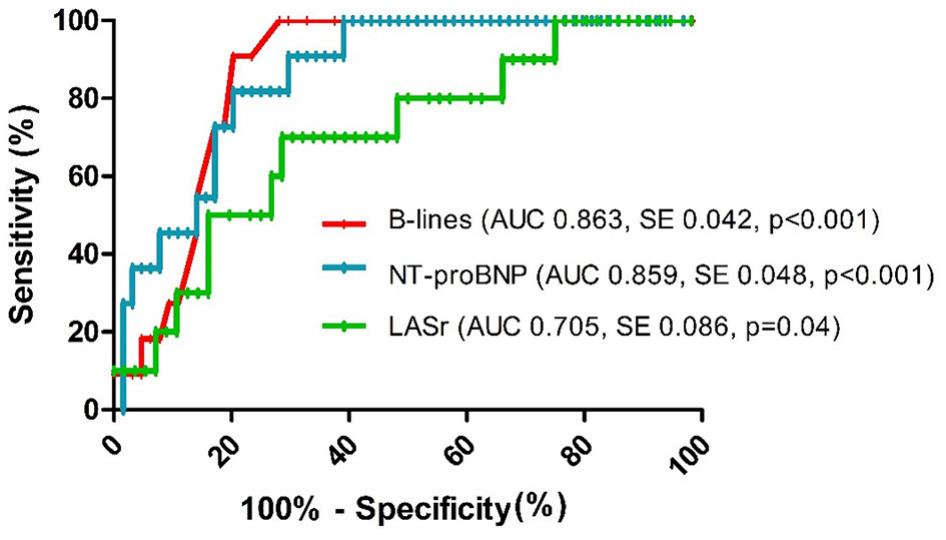
ROC curves for the prediction of endpoint events (AUC, Area under the curve; SE, standard error).

**Figure 4:**
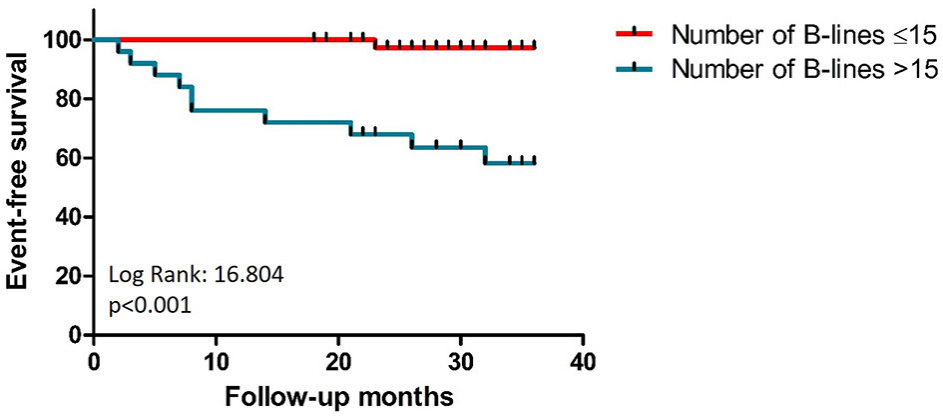
Comparison of Kaplan-Meier curves for patients with and without B-lines >15.

The feasibility of lung ultrasound is 100%, and the mean duration of the examination was 2.5 ± 0.47 min. We found a strong correlation between the number of B-lines and NT-proBNP levels and moderate correlation between B-lines and LASr (Figure 2). B-lines significantly correlated with estimated pulmonary artery systolic pressures (PASP; *r* = 0.471, *p* < 0.001) and left atrial volume index (LAVI; *r* = 0.243, *p* < 0.05), too. The performance of the number of B-lines in the prediction of HF events was similar to the performance of NT-proBNP levels (Figure 3), with the best cut-off value at 16 B-lines (sensitivity 91%, specificity 79%), which corresponds with the widely used cut-off for moderate PC (15). LASr predictive value was weaker (Figure 3), with the best cut-off at 13.75% (sensitivity 71.4%, specificity 70%). The feasibility of the LASr measurements was 92%. During the 26 [22,32] months follow up we detected 11 events: 4 patients were treated at an emergency department for an acute HF episode, 2 patients were admitted to the cardiology ward due to severe HF symptoms, 3 patients needed ambulatory intensification of loop diuretic treatment due to worsening of HF symptoms and 2 patients died (1 unknown cause, 1 patient during HF event). Having **>**15 B-lines significantly increased the risk of the endpoint events, and during the multivariate analysis, proved it to be an independent predictor of endpoint events (Table 2). The event-free survival was significantly worse among patients with >15 B-lines (*p* < 0.001, Log Rank: 16.804). The probability of cumulative event-free survival at 20 and 40 months in patients with ≤ 15 B-lines was 100 and 97.3%, respectively, while in patients with >15 B-lines it was 72% at 20 and 58.2% at 40 months (Figure 4).

**Table 2:** Cox regression analysis demonstrating the prognostic capacity of the predictor parameters.

**Parameters**	**Univariate analysis**		**Multivariate analysis**	
	***p***	**Hazard ratio**	***p***	**Hazard ratio**
Diabetes mellitus	–	–	–	–
Hyperlipidemia	0.024	5.96	–	–
Digoxin	–	–	–	- -
NT-proBNP	0.008	1.001	–	–
LASr	–	–	–	–
S′	0.029	0.769	–	–
DCT	0.023	0.986	–	–
B-lines > 15	0.004	20.956	0.01	15.473

*NT-proBNP, N-terminal (NT)-prohormone B type natriuretic peptide; LASr, Left Atrial Reservoir Strain; DCT, E wave deceleration time; S′, systolic myocardial velocity measured with Tissue Doppler Imaging*.

## Discussion

In our study of newly diagnosed HFpEF patients, having more than 15 B-lines at the time of diagnosis was highly suggestive of a worse prognosis and performed better in predicting HF events than NT-proBNP and the other clinical and echo parameters.

Assessing the number of B-lines is a simple, radiation-free and easily accessible method to estimate PC with 100% feasibility and short examination time (15, 28). Due to its advantages, a lot of data have been gathered until now about its potential use in different clinical settings. B-lines correlate with several clinical and echocardiographic parameters (16–18). We also found that the number of B-lines has a relationship with LA volume and estimated systolic pulmonary artery pressures. In our study, B-lines showed a close relationship with left atrial dysfunction represented by decreased LASr, which is a new observation. The commonly used cut-off value is >15 for moderate and >30 B-lines for severe congestion summing B-lines from 28 anterolateral lung areas (15). Examining HFrEF outpatients, Miglioranza et al. found the best cut-off value to be at 15 B-lines (18). Another study with pre-discharge HF patients confirmed this cut-off irrespective of EF (29), which correlates with our findings since the best cut-off value was at 16 B lines in our cohort. B-lines also have an exceptional prognostic value, shown in patients with HF (19–21, 29–31). After a 1-year follow-up in dyspneic patients, an increased number of B-lines was associated with a higher hospitalization rate with a best cut-off at 6 B-lines (8 sector LUS) (32). Measurement of PC at discharge provides prognostic information for patients with either HFpEF (33, 34) or HFrEF (34). Rueda-Camino et al. found significantly more hospital readmissions and HF deaths among patients with at least 15 B-lines (using the 28-segment LUS method) (33). According to Palazzuoli et al., B-lines ≥22 at discharge was associated with higher HF rehospitalization rate and all-cause mortality, and that prognostic value was similar in both HFpEF and HFrEF patients (34). The learning curve is very short for the acquisition of B-lines (35). With handheld ultrasound machines, this diagnostic tool could aid general practitioners as a point-of-care test.

Natriuretic peptides are frequently used biomarkers for diagnosis, risk stratification and therapeutic decision making in HF; however, HFpEF is a very heterogeneous disease, which makes both setting up the diagnosis and estimating prognosis more difficult. BNP and NT-proBNP are recognized outcome-predicting factors in acute HF regardless of EF (36). However, many studies suggested that its prognostic value remains controversial. The discharge NT-proBNP levels predicted outcomes similarly in HFpEF and HFrEF; however, Salah et al. concluded that comorbidities contribute more to prognosis in patients with HFpEF with lower NT-proBNP levels than in patients with HFrEF (10). Another pitfall of natriuretic peptide-based prognosis estimation is that its cut-off may depend on gender, age, body mass index, presence or absence of atrial fibrillation and renal failure (37–40). Eriksson et al. described significantly higher NT-proBNP values among HFmrEF and HFpEF patients in the event cohort for all-cause mortality, but the standard deviations were very high at 1, 3, and also 5 years (for HFpEF patients the means ± SD were 5,035.9 ± 5,630.3/3,785.1 ± 4,647.7/3,493.2 ± 4,365.5 ng/l), which reduces the prognostic utility of NT-proBNP in clinical practice (41). The levels are generally higher in patients presenting with acute HF than in patients with chronic HF (42). Additionally, the thicker myocardial wall, which is commonly seen in HFpEF, can normalize the wall stress, so even in the case of invasively proven HFpEF, the natriuretic peptide levels can be below the widely used threshold (43). These weaknesses are not characteristic of B-lines because PC is a frequent and almost universal pathophysiological phenomenon in patients with HF. It is not influenced by age, gender or body mass index. B-lines have diagnostic and prognostic utility without being affected by comorbidities except for diseases that involve lung parenchyma.

In the last 10 years, LA deformation imaging has become more and more widespread in research and daily routine. The LA is closely connected with the pulmonary venous system, and its dysfunction may play an essential role in the pathophysiology of PC. LA pressure increases to augment LV filling, resulting in pulmonary and systemic venous congestion. The LASr is an easy to measure and reproducible parameter, and it is now widely recognized that it has diagnostic and prognostic value regardless of EF (44, 45). LASr correlates well with diastolic dysfunction (46, 47) and the invasively measured LV filling pressure (48, 49), which plays a leading role in the pathophysiology of HFpEF, and it may have a prognostic value, too (45, 50). In patients with chronic HFrEF, LASr ≤ 12.9% showed a much worse outcome than higher strain values (44). In another study enrolling post-hospitalized HFpEF patients, LASr was an independent predictor of cardiovascular events, and LASr <31.2% was associated with significantly worse event-free survival (45). In our current study, the LASr was significantly reduced in the event group compared to those without any events (14.46 ± 6.98% vs. 20.71 ± 8.84%). It correlated well with both NT-proBNP and the number of B-lines. Still, we could not prove it to be an independent prognostic factor in HFpEF. The possible explanation is that we also included patients with atrial fibrillation. Park et al. found in 3,818 patients that the lowest tertile of the peak atrial longitudinal strain is predictive in acute HF patients regardless of EF; however, when subgroup analysis was performed, LASr did not show predictive value in the AF population (51). These results also emphasize the advantage of B-lines, which are not influenced by atrial fibrillation.

Finally, several score systems exist to estimate the risk of HFpEF patients, but until now, none of them has been recommended by guidelines. The widely used H2FPEF and HFA-PEFF scores were designed as diagnostic tools and were validated only on hospitalized, acute HFpEF population. The H2FPEF score might be a potentially useful marker for the prediction of cardiovascular and HF-related events in HFpEF patients (5, 52). Sotomi et al. found that the HFA-PEFF score is an excellent diagnostic tool, and it also has a practical prognostic value (4). Parcha et al. concluded that HFA-PEFF and the H2-FPEF scores are reliable diagnostic tools; however, their prognostic utility requires further validation (6). The mentioned score systems incorporate echocardiographic parameters like EF, E/e′, estimated systolic pulmonary pressure, left atrial volume index, relative wall thickness, and left ventricular mass index. Measurement of these parameters needs a comprehensive echocardiographic examination, which is time-consuming, requires an expert and might not be readily available. On the other hand, B-line assessment is simple and feasible, takes only a few minutes, and allows to visualize PC, which is the main pathophysiological change and the direct cause of symptoms in HF.

Limitations: As this is a single center study, the study population was relatively small, and the number of events was limited (*n* = 11). However, our results are consistent with previous studies on larger populations demonstrating the value of B-lines in patients with HFpEF and in patients with dyspnea and all spectrum of resting EF (53, 54). We showed the prognostic value of B-lines at rest. However, PC is a dynamic variable, and one-third of patients with HFpEF (55) or HFrEF (56) without B-lines at rest will develop PC during exercise. The number of B-lines during stress outperforms the prognostic value of B-lines at rest in patients with HFpEF (53, 55), in patients with HFrEF (56) and in consecutive patients with the full range of underlying resting ejection fraction (57). Therefore, our current study protocol has been adapted and currently includes a dynamic evaluation of B-lines also during stress in the framework of stress echo 2020 multicenter study (54). Many diseases which could have had an impact on the number of B-lines, the echocardiographic findings or the patient's heart failure symptoms were excluded at screening. The study population still remained quite heterogeneous; however, this heterogeneity reflects the circumstances under which the prognosis is estimated in everyday practice. We used a 28-zone protocol, which is more time-consuming than the simplified protocols, but performing the lung ultrasound only took a few minutes. The detection of B-lines does not necessarily imply their cardiogenic origin since pulmonary fibrosis and non-cardiogenic pulmonary oedema may also result in the presence of B-lines; however, we were applying strict exclusion criteria, so our study population did not have the mentioned etiological backgrounds.

## Conclusion

HFpEF is common, and prevalence is increasing. A feasible and straightforward diagnosis is crucial. The visualization of PC by LUS in HFpEF patients may contribute to the adequate diagnosis in the ambulatory setting. According to our results, it seems that B-lines in this population are good prognostic indicators. Also, it can be a powerful help in everyday practice to put our most vulnerable HFpEF patients in the spotlight. More studies with larger patient numbers are needed to confirm these findings and find lung ultrasound's proper place among the currently used diagnostic and prognostic score systems.

## Data Availability Statement

The raw data supporting the conclusions of this article will be made available by the authors, without undue reservation.

## Ethics Statement

The studies involving human participants were reviewed and approved by Regional Human Biomedical Research Ethics Committee of the University of Szeged. The patients/participants provided their written informed consent to participate in this study.

## Author Contributions

GÁ contributed to the conception and design of the study, data acquisition, analysis and interpretation, work drafting, gave final approval of the submitted version, and taking responsibility for the integrity of the work. BM-I contributed to data analysis and interpretation and work drafting and final approval of the present version. IS contributed to data analysis and interpretation and gave final approval of the submitted version. LG and AV contributed to the conception and design of the study, data interpretation, and work drafting also gave final approval of the version to be published. NP-N and MM contributed to the statistical revision of the paper and work drafting and gave final approval of the submitted version. EP contributed to the data interpretation and work drafting and gave final approval of the submitted version. All authors contributed to the article and approved the submitted version.

## Conflict of Interest

The authors declare that the research was conducted in the absence of any commercial or financial relationships that could be construed as a potential conflict of interest.

## Publisher's Note

All claims expressed in this article are solely those of the authors and do not necessarily represent those of their affiliated organizations, or those of the publisher, the editors and the reviewers. Any product that may be evaluated in this article, or claim that may be made by its manufacturer, is not guaranteed or endorsed by the publisher.

## Abbreviations

HFpEF: Heart failure with preserved ejection fraction
HFrEF: heart failure with reduced ejection fraction
HF: heart failure
EF: ejection fraction
LV: left ventricle
LA: left atrium
PC: pulmonary congestion
LUS: lung ultrasound
TTE: transthoracic echocardiogram
COPD: chronic obstructive pulmonary disease
eGFR: estimated glomerular filtration rate
LASr: left atrial reservoire strain
NT-proBNP: N-terminal pro B-type natriuretic peptide.
